# Ischemic Stroke Risk Assessment by Multiscale Entropy Analysis of Heart Rate Variability in Patients with Persistent Atrial Fibrillation

**DOI:** 10.3390/e23070918

**Published:** 2021-07-19

**Authors:** Ghina Chairina, Kohzoh Yoshino, Ken Kiyono, Eiichi Watanabe

**Affiliations:** 1 Graduate School of Science and Technology, Kwansei Gakuin University, Sanda 669-1337, Japan; dtf87669@kwansei.ac.jp; 2 Faculty of Mathematics and Natural Sciences, Universitas Padjadjaran, Sumedang 45363, Indonesia; 3 Graduate School of Engineering Science, Osaka University, Toyonaka 560-8531, Japan; kiyono@bpe.es.osaka-u.ac.jp; 4 Department of Cardiology, Fujita Health University Bantane Hospital, Nagoya 454-8509, Japan; enwatan@fujita-hu.ac.jp

**Keywords:** atrial fibrillation, ischemic stroke, heart rate variability, multiscale entropy

## Abstract

It has been recognized that heart rate variability (HRV), defined as the fluctuation of ventricular response intervals in atrial fibrillation (AFib) patients, is not completely random, and its nonlinear characteristics, such as multiscale entropy (MSE), contain clinically significant information. We investigated the relationship between ischemic stroke risk and HRV with a large number of stroke-naïve AFib patients (628 patients), focusing on those who had never developed an ischemic/hemorrhagic stroke before the heart rate measurement. The ${\mathrm{CHA}}_{2}{\mathrm{DS}}_{2}-\mathrm{VASc}$ score was calculated from the baseline clinical characteristics, while the HRV analysis was made from the recording of morning, afternoon, and evening. Subsequently, we performed Kaplan–Meier method and cumulative incidence function with mortality as a competing risk to estimate the survival time function. We found that patients with sample entropy ($S_{E}^{\left(s\right)}$) ≥ 0.68 at 210 s had a significantly higher risk of an ischemic stroke occurrence in the morning recording. Meanwhile, the afternoon recording showed that those with $S_{E}^{\left(s\right)}$ ≥ 0.76 at 240 s and $S_{E}^{\left(s\right)}$ ≥ 0.78 at 270 s had a significantly lower risk of ischemic stroke occurrence. Therefore, $S_{E}^{\left(s\right)}$ at 210 s (morning) and 240 s ≤ s ≤ 270 s (afternoon) demonstrated a statistically significant predictive value for ischemic stroke in stroke-naïve AFib patients.

## 1. Introduction

Atrial fibrillation (AFib) is the most common arrhythmia in elderly patients and is a known risk factor for stroke [1]. In patients without underlying rheumatic mitral valve disease, AFib is associated with an almost five-fold increase in the risk of stroke after adjusting for other risk factors [2]. In addition, strokes associated with AFib are often more disabling and fatal than strokes not associated with AFib, as most strokes associated with AFib are cardioembolic [3,4,5,6]. Consequently, the ability to predict ischemic stroke risk in patients with AFib is becoming an urgent issue, as many studies have reported a strong association between the two. The global focus on reducing stroke risks in AFib warrants an expanded understanding of the epidemiology, risk factors, determinants, and outcomes of stroke and other vascular conditions that threaten brain health [7]. Using the clinical characteristic information of patients, such as cardiovascular disease, prior stroke history, age, and sex, the ${\mathrm{CHA}}_{2}{\mathrm{DS}}_{2}-\mathrm{VASc}$ score is a widely used stroke predictor in patients with AFib [8].

Heart rate variability (HRV), defined as the interbeat intervals in normal sinus rhythm (without cardiac arrhythmia such as AFib), is closely related to the autonomic nervous system (ANS) activity [9]. This means the HRV characteristics can be used as a marker of ANS activity. By contrast, interbeat intervals, described as interventricular responses in AFib, have been considered to exhibit a rather white-noise-like behavior, independent of ANS activity [10]. However, recent studies of HRV in patients with AFib have demonstrated that HRV is not completely random, and still contains significant prognostic information concerning the risk of mortality and ischemic stroke [11].

There are limited studies regarding the HRV features of patients with AFib before an ischemic stroke event. Previous studies have reported the use of multiscale entropy (MSE) analysis of HRV as a predictor of ischemic stroke in patients with AFib [12,13]. Watanabe et al. [12] demonstrated that patients with AFib with a higher value of sample entropy at a time scale of 90 s ≤ *s* ≤ 300 s were more likely to develop ischemic stroke. Furthermore, MSE profiles are affected by multiscale characterizations, such as time correlation and probability distribution characteristics. Matsuoka et al. [13] reported that information entropy as the probability distribution characteristic at scales of 2 s and greater dominantly contribute to the risk assessment of ischemic stroke events. However, previous studies did not focus on stroke-naïve patients with AFib. The term “stroke-naïve” in this study is used to define patients with AFib who had not experienced an ischemic or hemorrhagic stroke before the heart rate measurement, including intracranial hemorrhage. Furthermore, the sample size of patients included in previous studies was small (173 patients). Thus, our goal was to clarify the association between HRV profiles and ischemic stroke risk with a higher number of stroke-naïve patients with AFib.

## 2. Materials and Methods

### 2.1. Patient Selection

**Figure 1:**
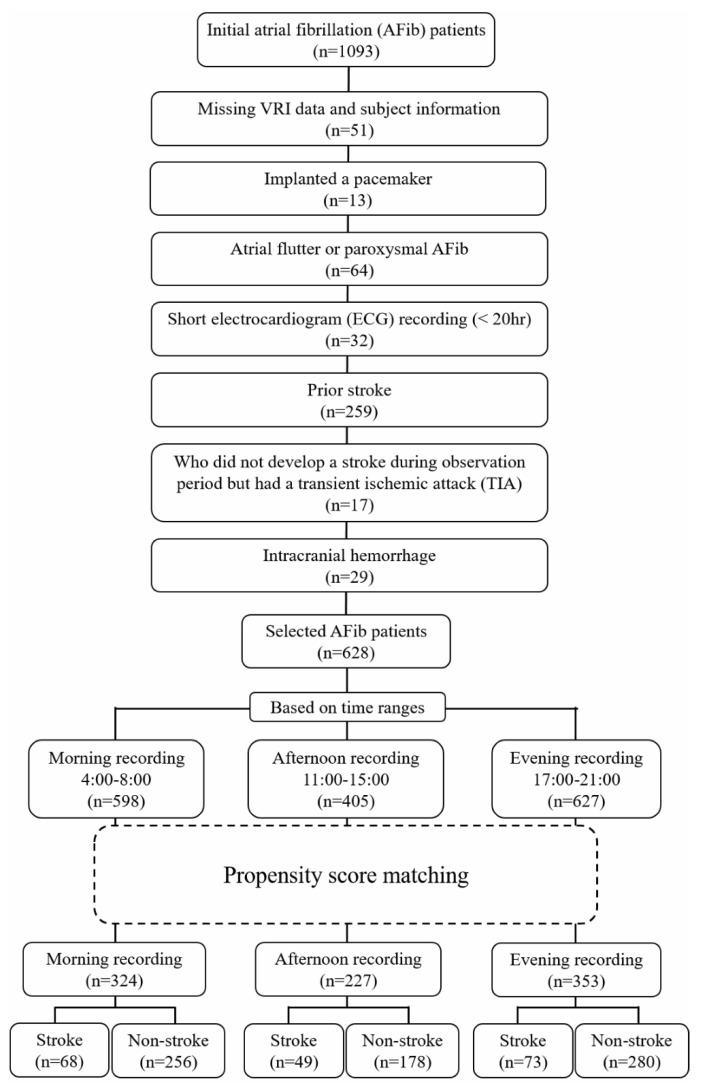
Patient selection flowchart.

Data pertaining to clinical characteristics and 24-h electrocardiogram (ECG) of 1093 patients with AFib were recorded from January 2005 to December 2013 at the Fujita Health University Hospital, Aichi, Japan. We excluded patients with certain criteria, such as (1) those with missing HRV data and missing or incorrect subject information; (2) those with a pacemaker; (3) those with atrial flutter or paroxysmal AFib; (4) those whose length of ECG recording was shorter than 20 h; (5) those who had a prior stroke; (6) those who did not develop an ischemic stroke during the observation period, but had a transient ischemic attack (TIA); and (7) those who had an intracranial hemorrhage. The remaining patients were then separated into two groups: (1) the ischemic stroke and (2) the non-ischemic stroke groups, which comprised patients who developed and did not develop an ischemic stroke during the follow-up years, respectively. Thereafter, the selected 628 stroke-naïve AFib patients were grouped based on their availability of morning (04:00 a.m.–08:00 a.m.), afternoon (11:00 a.m.–15:00 p.m.), and evening (17:00 p.m.–21:00 p.m.) recordings. A flowchart of patient selection is presented in Figure 1. We did not analyze the data during midnight hours, as sleep disorders (such as obstructive sleep apnea syndrome) might affect the sample entropy result [14]. To reduce the effect of confounding factors, we applied propensity score matching to the remaining patients with covariates as follows: age; body mass index (BMI); sex; inpatient/outpatient department; underlying diseases such as hypertension, coronary artery disease, heart failure, diabetes, dilated cardiomyopathy, and hypertrophic cardiomyopathy; and treatment with digitalis and warfarin. Propensity score matching was performed by matching one-on-four without replacement and a caliper of 0.2 of the standard deviation of the logit of the propensity score. In this study, the HRV time series is referred to as ventricular response interval (VRI) in AFib patients. VRI recordings were extracted automatically from the 24-h Holter electrocardiograms (ECGs) (Nihon Kohden, Tokyo, Japan), then interpolated linearly and resampled at 4 Hz. This research was approved by the ethics committee of Fujita Health University (approval No. HM17-232) for data measurement and Kwansei Gakuin University ethics committee (approval No. KG-IRB-18-02) for data analysis, and conformed to the principles outlined in the Declaration of Helsinki. For the use of data, the opt-out recruitment was approved by the Fujita Health University.

### 2.2. $CHA_{2}DS_{2}-VASc$ Score

We calculated the ${\mathrm{CHA}}_{2}{\mathrm{DS}}_{2}-\mathrm{VASc}$ score, where the acronym of “V” for vascular disease was replaced by coronary artery disease because of the absence of myocardial infarction, peripheral artery disease, and aortic plaque data. This score consists of 1 point for participants who had a congestive heart failure (C), 1 point for hypertension (H), 2 points for those with the age of 75 years or older ($A_{2}$), 1 point for diabetes (D), 2 points for prior stroke or TIA ($S_{2}$), 1 point for coronary artery disease as the vascular disease (V), 1 point for those with the age range from 65 to 74 years old (A), and 1 point for female participant (S).

### 2.3. Analysis of VRI

We calculated the conventional linear indices of VRI in the time-domain, including the mean VRI and standard deviation of VRI (SDVRI). We also applied non-linear analysis to better express the complex nature of VRI, as non-linear analysis is considered to be less dependent on the pre-processing of a recording [15,16,17,18,19]. Long-range correlation properties were evaluated using detrended fluctuation analysis (DFA) [20,21,22]. This was estimated by the scaling exponent α in $F\left(s\right)\;\sim \;s^{\alpha }$, where F(s) is defined as the square root of mean-square deviations around a linear trend averaged over segments with length *n* of integrated time series.

To measure the irregularity of the VRI time series $x_{i}$, multiscale entropy analysis (MSE) and multiscale characterizations of the time series were calculated. MSE was performed in two steps [10]: (1) coarse-graining of the VRI time series, and (2) sample entropy measurement of each coarse-grained time series. Given a one-dimensional discrete VRI time series $\left\{x_{1},\;x_{2},\ldots ,x_{N}\right\}$, the coarse-grained time series $\left\{y_{j}^{\left(s\right)}\right\}$ was calculated as follows:

(1) $$y_{j}^{\left(s\right)}=\frac{1}{v}\overset{jv}{\underset{i=\left(j-1\right)v+1}{\sum }}x_{i},$$

where $y_{j}^{\left(s\right)}$ is the mean value in *j*-th non-overlapping segment with length *v*, and $1\leq j\leq \frac{N}{v}$. The length *v* divided by the resampling frequency of 4 Hz is called the time scale, which is denoted as *s* = *v*/4. The range of the time scale *s* (seconds) was set to a range of 1 s ≤ *s* ≤ 300 s. In the next step, we calculated the sample entropy $S_{E}^{\left(s\right)}$ for each time scale *s* by the following:

(2) $$S_{E}^{\left(s\right)}\left(m,r,\bar{N}\right)=-\ln\frac{\sum_{i=1}^{\bar{N}-m-1}n_{i}^{\left(s\right)}\left(m+1,r\right)}{\sum_{i=1}^{\bar{N}-m}n_{i}^{\left(s\right)}\left(m,r\right)},$$

where *m* is the subseries length, *r* is the similarity tolerance, and $\bar{N}$ is the length of the VRI time series. Furthermore, $n_{i}^{\left(s\right)}\left(m,r\right)$ and $n_{i}^{\left(s\right)}\left(m+1,r\right)$ represent the number of vectors that match the *i*th template of length *m* and *m* + 1, respectively, which satisfies *r*. We set the value of *m* to 2 and *r* to 0.15$\sigma_{x}$, where $\sigma_{x}$ is the standard deviation of the resampled VRI time series. The sample entropy is the negative natural logarithm of the conditional probability that two subseries similar for *m* points remain similar for *m* + 1, where self-matches are not included in calculating the probability [23,24]. In addition, the sample entropy is influenced by the time correlation and the probability distribution characteristics. Therefore, we calculated the autocorrelation coefficient at lag *τ* = 1 to quantify the time correlation characteristic by the following:

(3) $${\hat{R}}^{\left(s\right)}\left(\tau \right)=\frac{1}{\bar{N}-\tau }\overset{\bar{N}-\tau }{\underset{i=1}{\sum }}\left(y_{i}^{\left(s\right)}\right)\left(y_{i+\tau }^{\left(s\right)}\right).$$

We also calculated Shannon’s information entropy to quantify the probability distribution characteristics by the following:

(4) $$H_{D}^{\left(s\right)}=-\overset{n_{s}}{\underset{i=1}{\sum }}p_{i}^{\left(s\right)}\ln p_{i}^{\left(s\right)},$$

where the probabilities {$p_{i}$} were calculated using the histogram-based probability density function of {$y_{j}^{\left(s\right)}$} with a fixed bin width of 0.15$\sigma_{x}$, and zero bins were not counted in $n_{s}.$ The autocorrelation coefficient at lag *τ* = 1 has a negative correlation with sample entropy, whereas information entropy has a positive correlation with the sample entropy. Then, we calculated the variance ratio as the ratio between the variance of the coarse-grained time series {$y_{j}^{\left(s\right)}$} and the original time series {$x_{i}$}, denoted as $\sigma_{s}^{2}/\sigma_{x}^{2}$.

All of the analyses were applied to the resampled VRI time series, and the time scale unit of the MSE profiles was in seconds instead of beats, in order to avoid it being affected by the cardiac rhythm (sinus or non-sinus) and heart rate [12].

### 2.4. Statistical Analysis

Quantitative data are presented as mean ± standard deviation (SD) for continuous variables, and as mean (frequency) for categorical variables. For continuous variables, the Mann–Whitney U test was conducted to analyze the significant differences between the two groups. We also calculated the Cohen’s d effect size to determine the size of the differences between the groups. For survival time analysis, we applied the Kaplan–Meier method to estimate the survival rate of ischemic stroke and the log-rank test as the statistical test. Furthermore, we performed a cumulative incidence function (CIF) to evaluate the VRI index prediction with mortality as a competing risk and Gray’s test to compare the CIF directly. Competing risk is an event whose occurrence precludes the occurrence of the primary event of interest [25]. In this study, the competing risk was mortality, and the primary event of interest was ischemic stroke occurrence. Receiver operating characteristic (ROC) curves were assessed to estimate the optimal cut-off value by plotting the true positive rate (sensitivity) against the false positive rate (1-specificity) at various cut-off values. The optimal cut-off value for the Kaplan–Meier survival time function analysis and CIF analysis was estimated using the value with the shortest distance to the (0,1) point of the ROC curves. Statistical tests were performed using R statistical software. A two-tailed *p*-value < 0.05 was considered significant.

## 3. Results

### 3.1. Patient Clinical Characteristics

Following patient selection (Figure 1), the morning, afternoon, and evening recordings consisted of 324 (68 with ischemic stroke and 256 without ischemic stroke), 227 (49 with ischemic stroke and 178 without ischemic stroke), and 353 (73 with ischemic stroke and 280 without ischemic stroke) patients, respectively. Those who had developed an ischemic stroke during the follow-up period were included in the ischemic stroke group, while those who did not were included in the non-ischemic stroke group. The event of ischemic stroke was recorded during the follow-up of 3.25 ± 2.89 years, 3.46 ± 3.11 years, and 3.51 ± 3.02 years for the morning, afternoon, and evening recordings, respectively.

Several differences in the baseline clinical characteristics between patients with AFib who had an ischemic stroke and those who did not were observed before the propensity score matching, including the ${\mathrm{CHA}}_{2}{\mathrm{DS}}_{2}-\mathrm{VASc}$ score (Table 1). After matching, no significant difference was found in the baseline clinical characteristics between patients with AFib who developed an ischemic stroke and those who remained stroke-naïve for each time range (Table 2 for the morning recording). The ${\mathrm{CHA}}_{2}{\mathrm{DS}}_{2}-\mathrm{VASc}$ score did not show any significant differences between the two groups in any time range.

### 3.2. Analysis of VRI

**Figure 2:**
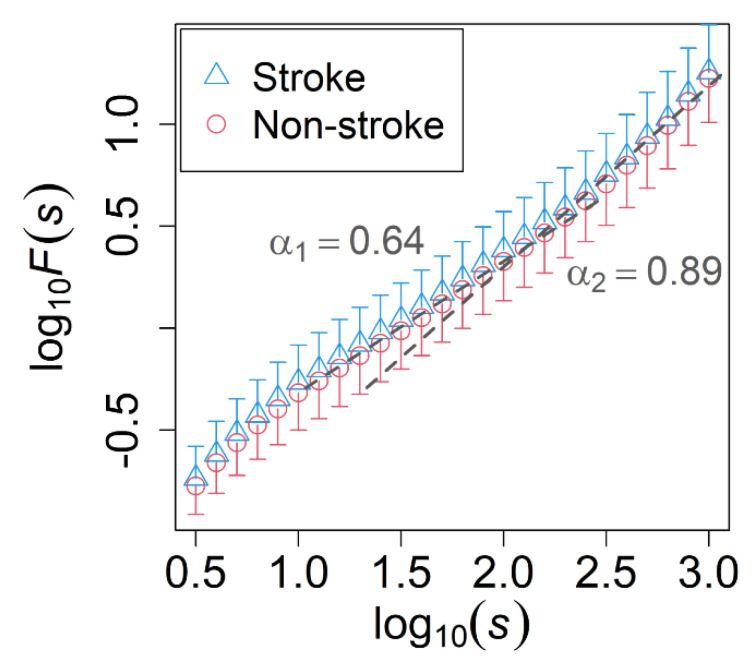
Fluctuation functions *F*(*s*) estimated by the detrended fluctuation analysis (DFA) of the morning recording. Comparison between stroke-naïve AFib patients who developed (blue triangle) and did not develop (red circle) an ischemic stroke during the observation period. The unit of *s* is seconds. Error bars represent the standard deviation, while dashed lines indicate the slopes with $\alpha_{1}$ = 0.64 and $\alpha_{2}$ = 0.89. No significant difference was found in the scaling exponents of DFA between the two groups.

There were no significant differences in the conventional linear indices of the time-domain, except for SDVRI in the evening recording range, which showed a borderline association with ischemic stroke (*p* = 0.04) (Table 3). The DFA of all of the time recordings showed that the scaling exponents was 0.64 at the scale less than approximately 100 s, which implied white-noise like behavior at small time scales, while it was 0.89 at the scale higher than 100 s, which implied the near-1/*f* fluctuation behavior at large time scales (morning recording range result in Figure 2). DFA scaling exponents did not show any significant difference between the two groups in small time scales and large time scales of all-time recordings. Furthermore, the statistical test of the MSE analysis showed that $S_{E}^{\left(s\right)}$ did not differ significantly between the two patient groups for any of the time ranges. Lastly, the variance ratio did not show any significant difference between the two groups of patients in any of the time ranges, demonstrating that the normalization of the time series was effective.

**Figure 3:**
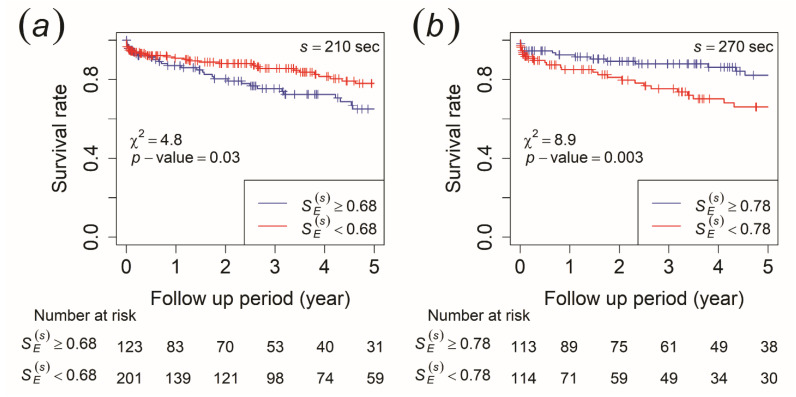
Kaplan–Meier survival curves for ischemic stroke occurrence. The patients were stratified by (**a**) $S_{E}^{\left(s\right)}$ ≥ 0.68 (blue line) and $S_{E}^{\left(s\right)}$ < 0.68 (red line) at a time scale of 210 s of the morning recording and (**b**) $S_{E}^{\left(s\right)}$ ≥ 0.78 (blue line) and $S_{E}^{\left(s\right)}$ < 0.78 (red line) at a time scale of 270 s of the afternoon recording.

No significant difference in $S_{E}^{\left(s\right)}$ was found between the two groups using the Mann–Whitney U test. We performed Kaplan–Meier survival analysis to check whether $S_{E}^{\left(s\right)}$ would be able to reflect the survival rate of ischemic stroke in stroke-naïve AFib patients when the survival time for each patient was included. In this analysis, survival time was defined as the length of time starting from the heart rate measurement date to the time when one had an ischemic stroke occurrence in the ischemic stroke group or the end of the follow-up period for the non-ischemic stroke group. The cut-off value was determined by the closest value to the (0,1) point in the ROC curve. Using the log-rank statistical test, the morning recording showed that patients with $S_{E}^{\left(s\right)}$ ≥ 0.68 at the time scale of 210 s had a significantly lower survival rate of ischemic stroke than those with $S_{E}^{\left(s\right)}$ < 0.68 (Figure 3a). This implied that they had a higher risk of an ischemic stroke occurrence. For the afternoon recording, those with $S_{E}^{\left(s\right)}$ ≥ 0.76 at the time scale of 240 s and $S_{E}^{\left(s\right)}$ ≥ 0.78 at the time scale of 270 s had a significantly higher survival rate of ischemic stroke than those with $S_{E}^{\left(s\right)}$ < 0.76 and $S_{E}^{\left(s\right)}$ < 0.78, which indicated they had a lower risk of an ischemic stroke occurrence (the result for 270 s is shown in Figure 3b).

**Figure 4:**
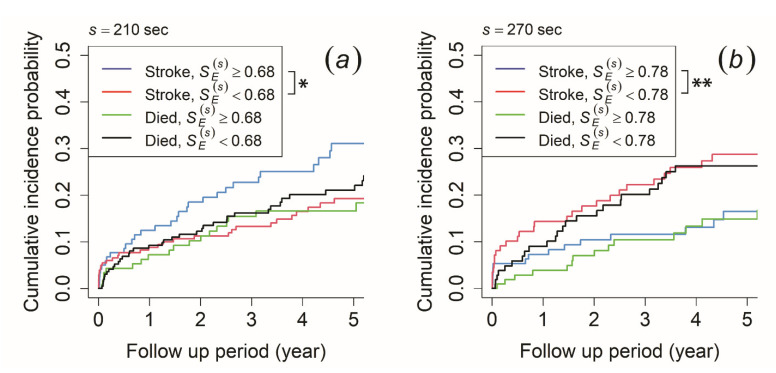
Cumulative incidence function for ischemic stroke occurrence and mortality. The patients were stratified by (**a**) $S_{E}^{\left(s\right)}$ ≥ 0.68 (blue and green line) and $S_{E}^{\left(s\right)}$ < 0.68 (red and black line) at a time scale of 210 s of the morning recording and (**b**) $S_{E}^{\left(s\right)}$ ≥ 0.78 (blue and green line) and $S_{E}^{\left(s\right)}$ < 0.78 (red and black line) at a time scale of 270 s of the afternoon recording. An asterisk (*) indicates *p*-value < 0.05, while double asterisks (**) indicates *p*-value < 0.01.

To verify this result, we set the mortality as a competing risk in the CIF method, while ischemic stroke was set as the primary outcome. For the morning recording, the CIF results revealed that patients with $S_{E}^{\left(s\right)}$ ≥ 0.68 at the time scale of 210 s had a significantly higher cumulative incidence probability of ischemic stroke than those with $S_{E}^{\left(s\right)}$ < 0.68, while no significant difference was observed in the risk of mortality (Figure 4a). In contrast, the afternoon recording showed that patients with $S_{E}^{\left(s\right)}$ ≥ 0.76 at the time scale of 240 s and $S_{E}^{\left(s\right)}$ ≥ 0.78 at the time scale of 270 s had a significantly lower cumulative incidence probability of ischemic stroke than those with $S_{E}^{\left(s\right)}$ < 0.76 and $S_{E}^{\left(s\right)}$ < 0.78, while no significant difference was observed for the risk of mortality (result for 270 s is shown in Figure 4b). Meanwhile, the cumulative incidence probability did not show any significant difference in both ischemic stroke risk and mortality risk for the evening recording.

**Figure 5:**
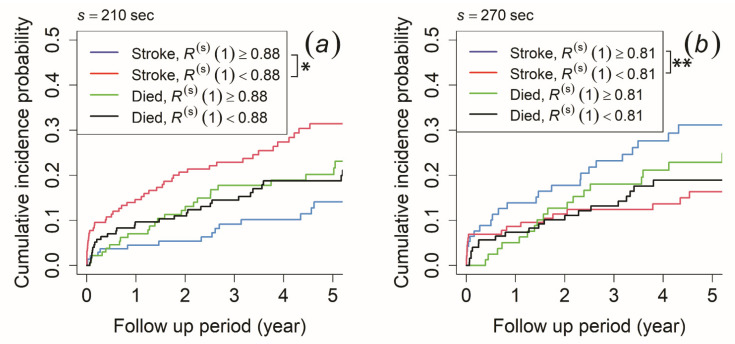
Cumulative incidence function for ischemic stroke occurrence and mortality. The patients were stratified by (**a**) autocorrelation coefficient at lag *τ* = 1 ($R^{\left(s\right)}\left(1\right)$) ≥ 0.88 (blue and green line) and $R^{\left(s\right)}\left(1\right)$ < 0.88 (red and black line) at a time scale of 210 s of the morning recording and (**b**) $R^{\left(s\right)}\left(1\right)$ ≥ 0.81 (blue and green line) and $R^{\left(s\right)}\left(1\right)$ < 0.81 (red and black line) at a time scale of 270 s of the afternoon recording. An asterisk (*) indicates *p*-value < 0.05, while double asterisks (**) indicates *p*-value < 0.01.

The MSE profiles were affected by characteristics such as the time correlation and probability distribution characteristics. To clarify which characteristic contributes to the ischemic stroke outcome and the difference in $S_{E}^{\left(s\right)}$, we performed survival time analyses to both the autocorrelation coefficient at lag *τ* = 1 ($R^{\left(s\right)}\left(1\right))$ to calculate the time correlation characteristic and the information entropy ($H_{D}^{\left(s\right)}$) for the probability distribution characteristic. The cut-off value was determined using the ROC curve. Based on the morning recording, patients with $R^{\left(s\right)}\left(1\right)$ < 0.88 at the time scale of 210 s had a significantly higher cumulative incidence probability of ischemic stroke than those with $R^{\left(s\right)}\left(1\right)$ ≥ 0.88, while no significant difference was observed in the risk of mortality (Figure 5a). For the afternoon recording, those with $R^{\left(s\right)}\left(1\right)$ < 0.82 at the time scale of 240 s and $R^{\left(s\right)}\left(1\right)$ < 0.81 at the time scale of 270 s had a significantly lower cumulative incidence probability of ischemic stroke than those with $R^{\left(s\right)}\left(1\right)$ ≥ 0.82 and $R^{\left(s\right)}\left(1\right)$ ≥ 0.81, while no significant difference was observed for the risk of mortality (the result for 270 s is shown in Figure 5b). The cumulative incidence probability did not show any significant difference in both ischemic stroke risk and mortality risk for the evening recording.

**Figure 6:**
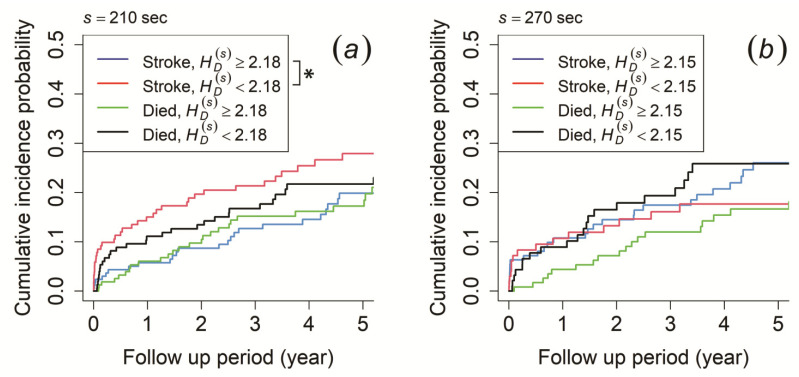
Cumulative incidence function for ischemic stroke occurrence and mortality. The patients were stratified by (**a**) information entropy ($H_{D}^{\left(s\right)}$) ≥ 2.18 (blue and green line) and $H_{D}^{\left(s\right)}$ < 2.18 (red and black line) at a time scale of 210 s of the morning recording and (**b**) $H_{D}^{\left(s\right)}$ ≥ 2.15 (blue and green line) and $H_{D}^{\left(s\right)}$ < 2.15 (red and black line) at a time scale of 270 s of the afternoon recording. An asterisk (*) indicates *p*-value < 0.05.

In terms of probability distribution characteristics, the morning recording showed that patients with $H_{D}^{\left(s\right)}$ ≥ 2.18 at the time scale of 210 s had a significantly higher cumulative incidence probability of ischemic stroke occurrence than those with $H_{D}^{\left(s\right)}$ < 2.18, while no significant difference was observed for mortality risk (Figure 6a). Meanwhile, the cumulative incidence probability did not show any significant difference for both ischemic stroke risk and mortality risk for the afternoon and evening recording (the result for 270 s of the afternoon recording is shown in Figure 6b).

## 4. Discussion

The present study demonstrated that sample entropy ($S_{E}^{\left(s\right)}$) at the time scale of 210 s of the morning recording and 240 s ≤ *s* ≤ 270 sec of the afternoon recording have a statistically significant prognostic value for ischemic stroke outcome in stroke-naïve AFib patients. Furthermore, the autocorrelation coefficient at lag *τ* = 1 as the time correlation characteristics of the MSE profiles might contribute to the difference in $S_{E}^{\left(s\right)}$.

We confirmed the presence of white noise-like fluctuations at shorter scales (less than approximately 100 s) and near-1/*f* fluctuations in longer scales (more than approximately 100 s) in the VRI time series of AFib patients (Figure 2) with $\alpha_{1}$ = 0.64 and $\alpha_{2}$ = 0.89, which is similar to the previous study [13]. Several findings have described the existence of white noise characteristics as an uncorrelated process (over short scales) and 1/*f* noise as a correlated process (over long scales) in the HRV of patients with AFib [11,26]. The dynamics of the regulatory process underlying the long-term component may be common between the HRV of a healthy subject and an AFib patient [11]. Thus, the time scales exhibiting near-1/*f* fluctuations were reported to reveal new information regarding the complexity of HRV, which could be measured by MSE analysis [10]. To evaluate the irregularity of ECG signals in the 1/*f* fluctuation area and the onset of a disease, sample entropy is considered to be an effective complex system analysis [27]. Furthermore, Ho et al. [28] also reported that the sample entropy calculated in the 1/*f* fluctuation area may serve as a significant predictor of mortality in patients with congestive heart failure. However, our Mann–Whitney U statistical test of $S_{E}^{\left(s\right)}$ did not show any significant difference between AFib patients who had an ischemic stroke outcome and those who remained stroke-naïve.

On that account, we intended to check the ability of $S_{E}^{\left(s\right)}$ to reflect ischemic stroke risk in stroke-naïve AFib patients by applying the Kaplan–Meier and CIF methods, where the length of time until an ischemic stroke event occurred was included. As a result, we found that patients with $S_{E}^{\left(s\right)}$ ≥ 0.68 at the time scale of 210 s of the morning recording had a higher risk of developing an ischemic stroke (Figure 3a). This result was consistent even when mortality was included as a competing risk in CIF analysis, where $S_{E}^{\left(s\right)}$ only reflected the risk of ischemic stroke outcome and not mortality (Figure 4a). This indicated that AFib patients who had a higher risk of an ischemic stroke occurrence were those whose VRI generated a higher degree of irregularity in the near-1/*f* fluctuation area of the morning recording. For the afternoon recording, we discovered that those with $S_{E}^{\left(s\right)}$ < 0.76 at the time scale of 240 s and $S_{E}^{\left(s\right)}\;$< 0.78 at the time scale of 270 s had a higher risk of developing an ischemic stroke (the result for 270 s is shown in Figure 3b). Even with a competing risk in the CIF analysis, this result remained consistent and showed that $S_{E}^{\left(s\right)}$ only reflected the risk of ischemic stroke outcome and not mortality (the result for 270 s is shown in Figure 4b). This suggests that patients with AFib whose VRI generated a lower degree of irregularity in the near-1/*f* fluctuation area had a higher risk of ischemic stroke for the afternoon recording. Thus, these results implied that $S_{E}^{\left(s\right)}$ in scales exhibiting near-1/*f* fluctuation demonstrated a statistically significant predictive value for ischemic stroke outcome in stroke-naïve AFib patients. Furthermore, the statistical test of $S_{E}^{\left(s\right)}$ by Mann–Whitney U test, which showed no significant difference between the two groups, maybe because of the length of survival time, which was not included in the analysis. Therefore, we discovered that $S_{E}^{\left(s\right)}$ was able to reflect the risk of an ischemic stroke event in stroke-naïve AFib patients when the survival time for each patient was included.

Our morning recording result was in agreement with the previous studies that $S_{E}^{\left(s\right)}$ at large time scales were higher in ischemic stroke patients than in non-ischemic stroke patients [12,13], while our afternoon recording result was similar to reports from other studies that demonstrated a decrease in nonlinear behavior of the heart rate, which is associated with the worsening of pathological states [29]. It has been reported that healthy participants generate more complex dynamics than diseased participants [30]. Therefore, our results confirmed that those with a decrease in the degree of irregularity were more likely to develop an ischemic stroke based on the afternoon recording. This may attributed to the loss of complexity in patients with unfavorable outcomes. Our research agreed with a previous finding showing that acute ischemic stroke patients with a significantly lower complexity were more likely to develop stroke-in-evolution than those with a higher complexity [31]. A similar result was also demonstrated, that higher values of the complexity index were significantly associated with favorable outcomes in patients [32]. Less complex dynamics were observed in patients with many states of disease compared with patients in healthy conditions [33,34]. This is because the information content is degraded as physiological systems become less complex. Furthermore, they become less adaptable and less able to cope with the exigencies of constantly changing environments [34,35]. Young healthy systems are the most complex and adaptive systems [30].

Based on the time range, there was a diurnal variation in $S_{E}^{\left(s\right)}$ found in this study. One possible explanation for this variation might be the presence of a circadian rhythm of $S_{E}^{\left(s\right)}$ in the 1/*f* fluctuation area of stroke-naïve AFib patients. A circadian rhythm is a physiological and behavioral cycle with a recurring periodicity of approximately 24 h, controlling a variety of biological processes, such as the sleep–wake cycle [36,37]. According to our results, patients who remained stroke-naïve had a lower degree of irregularity in the morning (indicated by lower $S_{E}^{\left(s\right)}$), followed by a higher degree of irregularity in the afternoon (indicated by higher $S_{E}^{\left(s\right)}$). Thus, we hypothesized that this circadian pattern of $S_{E}^{\left(s\right)}$ in scales larger than 100 s disappeared in patients who were at a higher risk of developing an ischemic stroke event.

As MSE profiles provide a possible characterization of the biosignal complexity [38], factors such as time correlation and probability distribution characteristics affect the results of $S_{E}^{\left(s\right)}$. Therefore, we applied both survival analyses to verify the factors contributing to the difference in $S_{E}^{\left(s\right)}$ in our study. The time correlation characteristic was calculated by autocorrelation coefficient at lag *τ* = 1 ($R^{\left(s\right)}\left(1\right)$), while the probability distribution characteristic was calculated by information entropy ($H_{D}^{\left(s\right)}$). As a result, we confirmed that $R^{\left(s\right)}\left(1\right)$ had a negative correlation with $S_{E}^{\left(s\right)}$ in the morning and afternoon recording. For the morning recording, patients with $R^{\left(s\right)}\left(1\right)$ < 0.88 at the time scale of 210 s had a higher risk of developing an ischemic stroke (Figure 5a). For the afternoon recording, those with $R^{\left(s\right)}\left(1\right)$ ≥ 0.82 at the time scale of 240 s and $R^{\left(s\right)}\left(1\right)$ ≥ 0.81 at the time scale of 270 s had a higher risk of developing an ischemic stroke (the result for 270 s is shown in Figure 5b). On the contrary, we found that $H_{D}^{\left(s\right)}$ had a negative correlation with the result of $S_{E}^{\left(s\right)}$ for the morning recording. The results showed that patients with $H_{D}^{\left(s\right)}$ < 2.18 at the time scale of 210 s had a higher risk of developing ischemic stroke (Figure 6a). Unlike the time correlation characteristic, $H_{D}^{\left(s\right)}$ was assumed to have a positive correlation. Thus, our result implied that only the time correlation characteristic might contribute to the difference in $S_{E}^{\left(s\right)}$, because $H_{D}^{\left(s\right)}$ had an adverse effect on $S_{E}^{\left(s\right)}$.

Matsuoka et al. [13] revealed that the probability distribution characteristic of MSE profiles in a wider range of scales of *s* ≥ 2 s is a useful measure for ischemic stroke risk assessment, while our result of $H_{D}^{\left(s\right)}$ did not show any significant difference between the two groups. Furthermore, the previous study did not find any significant difference in the time correlation characteristic between the two groups, while the present study found that $R^{\left(s\right)}\left(1\right)$ at longer time scales might contribute to $S_{E}^{\left(s\right)}$. The difference between our finding and previous studies could be as a result of two reasons: firstly, we had a larger number of participants in our study, which were selected by the propensity score matching based on several baseline clinical characteristics covariates to reduce the effect of confounding; secondly, we focused on “stroke-naïve AFib patients”, which means that only patients with AFib that had never developed any ischemic or hemorrhagic stroke before the heart rate measurement date were included.

This study had several limitations. The present study was an observational study with a cohort of Japanese patients at a single institution. This may have caused selection bias. To verify the findings of this study, further investigation with more heterogeneous participants (i.e., foreign patients) may eliminate the possibility of this bias and confirm whether the proposed hypothesis can be applied to a more diverse population. We also did not perform a comparison of the predictive performance in patients based on antithrombotic drug intake due to the limited data on the subject information. Moreover, the vascular disease in ${\mathrm{CHA}}_{2}{\mathrm{DS}}_{2}-\mathrm{VASc}$ score only consisted of coronary artery disease due to the absence of myocardial infarction, peripheral artery disease, and aortic plaque data. Furthermore, there were only a limited number of previous studies to interpret our results. Thus, the generalizability of our findings in each time range (morning, afternoon, and evening) may verify our proposed hypothesis, such as the presence of a circadian rhythm of $S_{E}^{\left(s\right)}$ in stroke-naïve AFib patients with a low risk of developing an ischemic stroke event.

## 5. Conclusions

Our study found that stroke-naïve AFib patients whose VRI generated a higher degree of irregularity in scales exhibiting near-1/*f* fluctuations had a higher risk of developing an ischemic stroke in the morning recording, while they had a lower risk of ischemic stroke outcome in the afternoon recording. We also found that the time correlation characteristic of the MSE profiles might contribute to the difference in $S_{E}^{\left(s\right)}$. In summary, $S_{E}^{\left(s\right)}$ at the time scale of 210 s for the morning recording and 240 s ≤ *s* ≤ 270 s for the afternoon recording demonstrated a statistically significant prognostic value for ischemic stroke outcome in stroke-naïve AFib patients. This finding may provide valuable information for improving ischemic stroke risk assessment in stroke-naïve patients with AFib.

## Figures and Tables

**Table 1:** Baseline clinical characteristics of patients before propensity score matching (628 selected patients with AFib).

Clinical Characteristics	Full Cohort (n = 628)		*p*-Value
	Ischemic Stroke (n = 74)	Non-Ischemic Stroke (n = 554)	
**Age, years**	72.73 ± 9.1	69.12 ± 11.87	0.01 *
BMI (kg/m^2^)	22.31 ± 3.72	22.89 ± 3.83	0.26
Female	23 (31)	180 (32)	0.81
Outpatient department	32 (43)	261 (47)	0.53
**Underlying disease**			
Hypertension	56 (76)	345 (62)	0.02 *
Coronary Artery Disease	34 (46)	204 (37)	0.13
Heart Failure	45 (61)	343 (62)	0.85
Diabetes	21 (28)	163 (29)	0.85
Dilated Cardiomyopathy	2 (3)	33 (6)	0.25
Hypertrophic Cardiomyopathy	2 (3)	11 (2)	0.68
**Medication**			
Warfarin	33 (45)	277 (50)	0.38
ACE Inhibitor, ARB	38 (51)	239 (43)	0.18
Ca-channel Blocker	5 (7)	44 (8)	0.72
Digitalis	15 (20)	176 (32)	0.04 *
${\mathrm{CHA}}_{2}{\mathrm{DS}}_{2}-\mathrm{VASc}$ score	3.73 ± 1.43	3.26 ± 1.59	0.01 *

Ischemic stroke: patients who developed an ischemic stroke during the observation period; non-ischemic stroke: patients who did not develop an ischemic stroke during the observation period; BMI: body mass index; ACE: angiotensin converting enzyme; ARB: angiotensin receptor blocker. Data are presented as the mean ± standard deviation (SD) or number and frequency. An asterisk (*) indicates *p*-value < 0.05.

**Table 2:** Baseline clinical characteristics of patients after propensity score matching for the morning recording range (04:00–08:00 a.m.).

Clinical Characteristics	Matched Subset (n = 324)		*p*-Value
	Ischemic Stroke (n = 68)	Non-Ischemic Stroke (n = 256)	
**Age, years**	72.41 ± 8.95	72.82 ± 10.44	0.67
BMI (kg/m^2^)	22.43 ± 3.73	22.29 ± 3.52	0.86
Female	22 (32)	81 (32)	0.91
Outpatient department	29 (43)	114 (45)	0.78
**Underlying disease**			
Hypertension	51 (75)	180 (70)	0.44
Coronary Artery Disease	29 (43)	112 (44)	0.87
Heart Failure	42 (62)	161 (63)	0.86
Diabetes	18 (26)	69 (27)	0.93
Dilated Cardiomyopathy	1 (1)	9 (4)	0.38
Hypertrophic Cardiomyopathy	2 (3)	5 (2)	0.61
**Medication**			
Warfarin	31 (46)	119 (46)	0.89
ACE Inhibitor, ARB	33 (49)	118 (46)	0.72
Ca-channel Blocker	5 (7)	22 (9)	0.74
Digitalis	15 (22)	58 (23)	0.91
${\mathrm{CHA}}_{2}{\mathrm{DS}}_{2}-\mathrm{VASc}$ score	3.69 ± 1.44	3.61 ± 1.5	0.79

Data are presented as the mean ± standard deviation (SD) or number and frequency. Abbreviations are the same as those in Table 1.

**Table 3:** Linear indices of heart rate variability in the time-domain.

Clinical Characteristics	Ischemic Stroke	Non-Ischemic Stroke	*p*-Value	Effect Size
**Morning recording (04:00–08:00 a.m.)**				
Mean VRI (s)	0.89 ± 0.22	0.84 ± 0.21	0.06	0.23
SDVRI (s)	0.2 ± 0.07	0.19 ± 0.06	0.07	0.23
**Afternoon recording (11:00 a.m.–14:00 p.m.)**				
Mean VRI (s)	0.79 ± 0.14	0.75 ± 0.15	0.07	0.23
SDVRI (s)	0.17 ± 0.05	0.16 ± 0.04	0.18	0.26
**Evening recording (17:00–21:00 p.m.)**				
Mean VRI (s)	0.81 ± 0.19	0.77 ± 0.17	0.09	0.19
SDVRI (s)	0.17 ± 0.05	0.16 ± 0.04	0.04 *	0.26

VRI: ventricular response interval; SDVRI: SD of VRI; s: seconds. Data are presented by as mean ± standard deviation (SD). An asterisk (*) indicates *p*-value < 0.05.

## Data Availability

The data are not publicly available due to privacy.
